# Editorial: Helping meet oral health needs in underserved communities

**DOI:** 10.3389/froh.2023.1257756

**Published:** 2023-07-31

**Authors:** Darien J. Weatherspoon, Michelle R. McQuistan, George W. Taylor, Keith A. Mays

**Affiliations:** ^1^ School of Dentistry, University of Maryland Baltimore, Baltimore, MD, United States; ^2^ College of Dentistry and Dental Clinics, The University of Iowa, Iowa City, IA, United States; ^3^ School of Dentistry, University of California, San Francisco, San Francisco, CA, United States; ^4^ School of Dentistry, University of Minnesota, Minneapolis, MN, United States

**Keywords:** oral health, disparities (health), underserved and unserved populations, dentistry, social determinants of health

**Editorial on the Research Topic** Helping meet oral health needs in underserved communities

The turn of the century was a seminal moment for oral health in the United States (U.S.). In the year 2000, Surgeon General Dr. David Satcher released the first ever Surgeon General's Report on oral health titled, “Oral Health in America: A Report of the Surgeon General” (1). In addition to its central message that oral health is inextricably linked to overall health conditions, including diabetes, cardiovascular disease, and quality-of-life; a major finding of the report was the revelation of long-standing disparities in oral disease burden experienced by many underserved communities in the U.S (1). Beyond merely describing these disparities, the report issued a call to action for advancement in research, policy, and practice initiatives to eliminate these inequities. Beyond the U.S., underserved communities in Asia and globally experience higher burdens of oral disease and oral health inequities (2–4).

Over the last 20 years, the oral health community and its stakeholders have taken heed to the call to action to address oral health inequities through several local and national initiatives. While these efforts are to be commended, inequities in oral health status still exist for many underserved groups, as highlighted by the more recent “Oral Health in America: Advances and Challenges,” a 2021 follow-up report to the original Surgeon General's report on oral health (5). Recognizing that evidence-based research is still needed to guide advancements in policies and practices aimed at ensuring equity in oral health for all, we sought a collection of articles to contribute to our research topic, “*Helping Meet Oral Health Needs in Underserved Communities*.” The primary objectives of this topic were to disseminate current research that is elucidating the increasingly complex drivers of oral health inequities and highlight innovative interventions aimed at improving the oral health of underserved populations.

Frameworks that highlight multi-level influences on oral health and inequities have been developed to guide interventions. For example, a model by Fisher-Owens demonstrates that a child's oral health is influenced not only by their individual factors, but also those of their family and community (6). Considering the need for multi-level interventions, Lee et al. contributed findings from their community health worker (CHW) intervention. As part of overall oral health promotion, CHWs attempted to address social determinants of health (SDOH) and psychosocial factors that often plague caregivers in underserved communities and are associated with poor oral health behaviors. Although the intervention did not result in positively changing psychosocial factors as hypothesized, the results are beneficial in informing future oral health interventions. The results demonstrated the importance of targeting SDOH in community-based interventions and highlighted the need for validated instruments that better capture psychosocial factors prevalent in underserved communities.

Other multi-level frameworks have been developed to conceptualize influences on oral health, such as one proposed by Lee and Divaris (7). Importantly, this framework is rooted in an ethical imperative to address upstream macro- and population-level policies and factors that can create conditions under which inequities can occur (7). Smith reinforced the ethical obligation oral health stakeholders have in addressing health inequities. He proposed a systems-oriented ethical decision-making framework that stakeholders can use to consider the social and structural determinants of health that disadvantaged populations face when making care delivery decisions. If implemented, use of this ethical framework could result in improved quality of oral health care delivery to underserved communities, ultimately improving oral health outcomes and reducing inequities.

Smith et al. discussed a model of dental education that could be used to increase students' awareness of the impact of SDOH on oral health inequities so they are better prepared to deliver holistic, patient-centered care to diverse communities as practitioners. Findings from reflection assignments demonstrated that foreign-trained dentists who participated in community-based dental education (CBDE) expressed having a better understanding of: 1) how social and structural determinants of health impact access to oral healthcare for underserved communities, and 2) their professional responsibilities in addressing these barriers. Therefore, utilizing CBDE during dental training can be a promising workforce strategy used to increase future dental practitioners' awareness of drivers of inequities, so they are better prepared to address them when they enter practice.

Individuals with developmental disabilities are an underserved group that is often overlooked. Previous research has shown that these individuals commonly have unmet dental needs, and face barriers in accessing preventive dental care (8–10). Atchison et al. presented findings on emergency department (ED) use among developmentally disabled (DD) adolescents with oral health complications. Using data from the National Survey of Children's Health, they found that DD adolescents with oral health problems were more likely than their counterparts to use the ED. They also found that not having a medical home was associated with increased ED use. Their results highlight the important role that integrated medical-dental health systems can play in reducing oral health inequities, particularly among those with DD and other medically-vulnerable groups.

More research is needed to better understand disparities in the provision of oral health services and treatment outcomes. Burns et al. analyzed state Medicaid administrative claims data to assess receipt of root canal therapy (RCT) and subsequent treatment outcomes in Medicaid insured children and adolescents by demographic factors. They found that African American and Hispanic children were less likely to receive multiple RCTs, less likely to have a permanent restoration placed after RCT, and more likely to have failed RCT treatment, compared to their White counterparts. While the results observed cannot be directly attributed to racism, the unequal treatment provision and outcomes based on race/ethnicity, highlight the need for more research, policies, and interventions aimed at mitigating structural racism and implicit bias, as a way to achieve equitable health outcomes. Addressing structural racism is a dental public health priority (11).

While there is still much work to be done to meet the oral health needs of vulnerable and underserved communities in the U.S. and globally, we believe that the diverse research we assembled provides important findings that can help inform future research, practice, and policies to achieve the vision of oral health equity for all.
